# Supplementary material for: The gastrin-releasing peptide/bombesin system revisited by a reverse-evolutionary study considering Xenopus
Source: Sci Rep. 2021 Jun 25;11:13315. doi: 10.1038/s41598-021-92528-x (PMC8233351; doi:10.1038/s41598-021-92528-x)
Supplement: Supplementary file 1 — Supplementary Information. [file 41598_2021_92528_MOESM1_ESM.pdf]

Supplementary Information

The gastrin-releasing peptide/bombesin system revisited by a reverse-evolutionary study considering *Xenopus*

Asuka Hirooka[1¶], Mayuko Hamada[1,2¶], Daiki Fujiyama[1], Keiko Takanami[1,#a], Yasuhisa Kobayashi[1,#b], Takumi Oti[1,#c], Yukitoshi Katayama[1], Tatsuya Sakamoto,[1] and Hirotaka Sakamoto[1,3]*

[1] Ushimado Marine Institute (UMI), Graduate School of Natural Science and Technology, Okayama University, Ushimado, Setouchi, Okayama 701-4303, Japan

[2] Zoological Institute, Christian-Albrechts University Kiel, Am Botanischen Garten 1-9, Kiel 24118, Germany

[3] Department of Physiology, Anatomy & Genetics, University of Oxford, South Parks Road, Oxford OX1 3PT, United Kingdom

[#a]Current Address: Mouse Genomics Resources Laboratory, National Institute of Genetics, Yata, Mishima, Shizuoka 411-8540, Japan

[#b]Current Address: Laboratory for Aquatic Biology, Department of Fisheries, Faculty of Agriculture, Kindai University, Nakamachi, Nara 631-8505, Japan

[#c]Current Address: Department of Biological Sciences, Faculty of Science, Kanagawa University, Hiratsuka, Kanagawa 259-1293, Japan

*Corresponding author

E-mail: hsakamo@okayama-u.ac.jp (HS)

[¶]These authors should be considered joint first author.

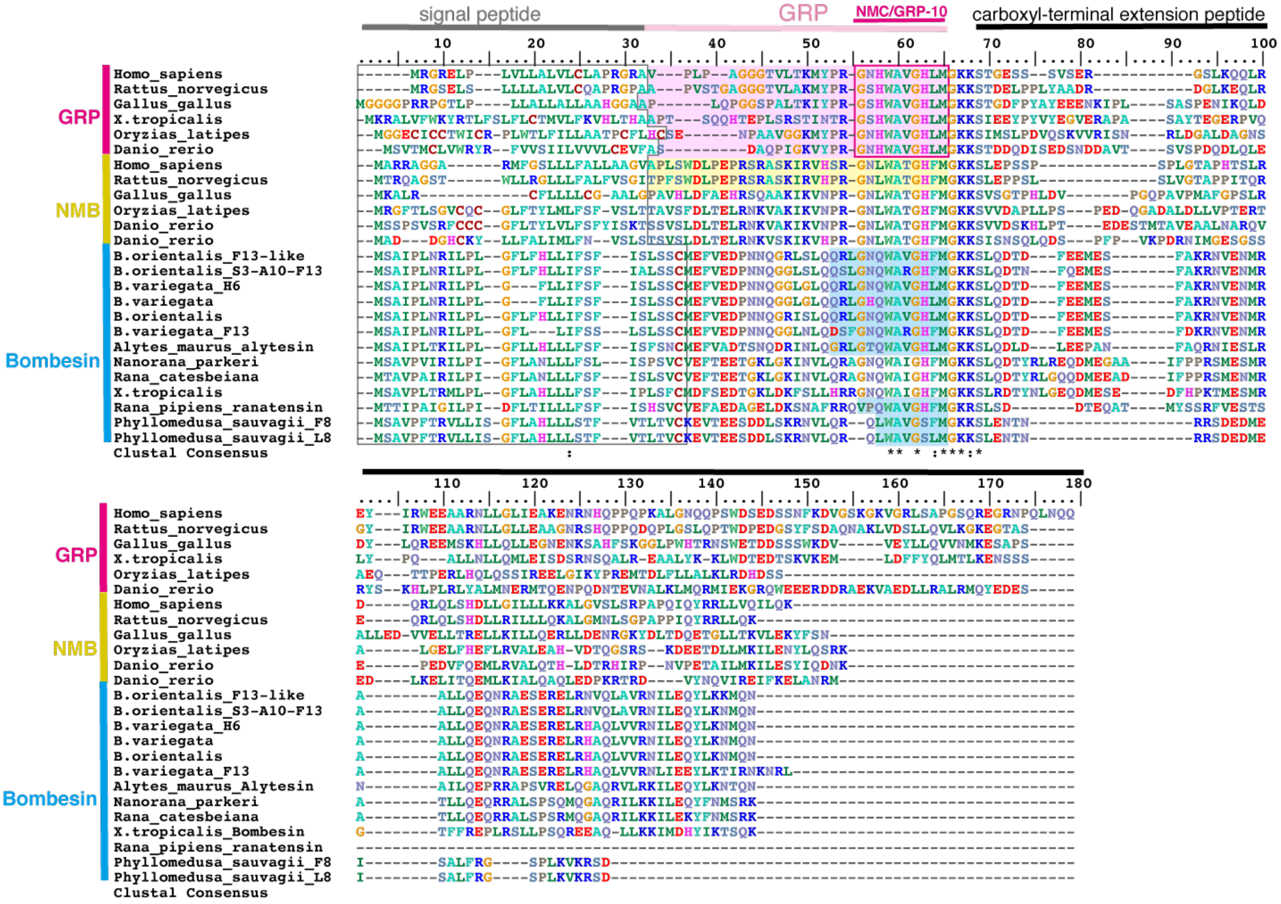

Supplementary Figure S1. Amino acid sequence alignment of gastrin-releasing peptide (GRP), neuromedin B (NMB) and bombesin. The signal peptide in each sequence by SignalP is shown in a gray frame. Bioactive GRP (mature GRP) and neuromedin C (NMC)/GRP-10 sites are highlighted in pink and a magenta box, respectively. Bioactive NMB in humans and rats and bombesin-like peptides previously found in frogs are highlighted in yellow and blue, respectively. Carboxyl-terminal extension peptide sites are represented by black line. IDs for the protein sequences used in this analysis are shown in Supplemental Table 1.

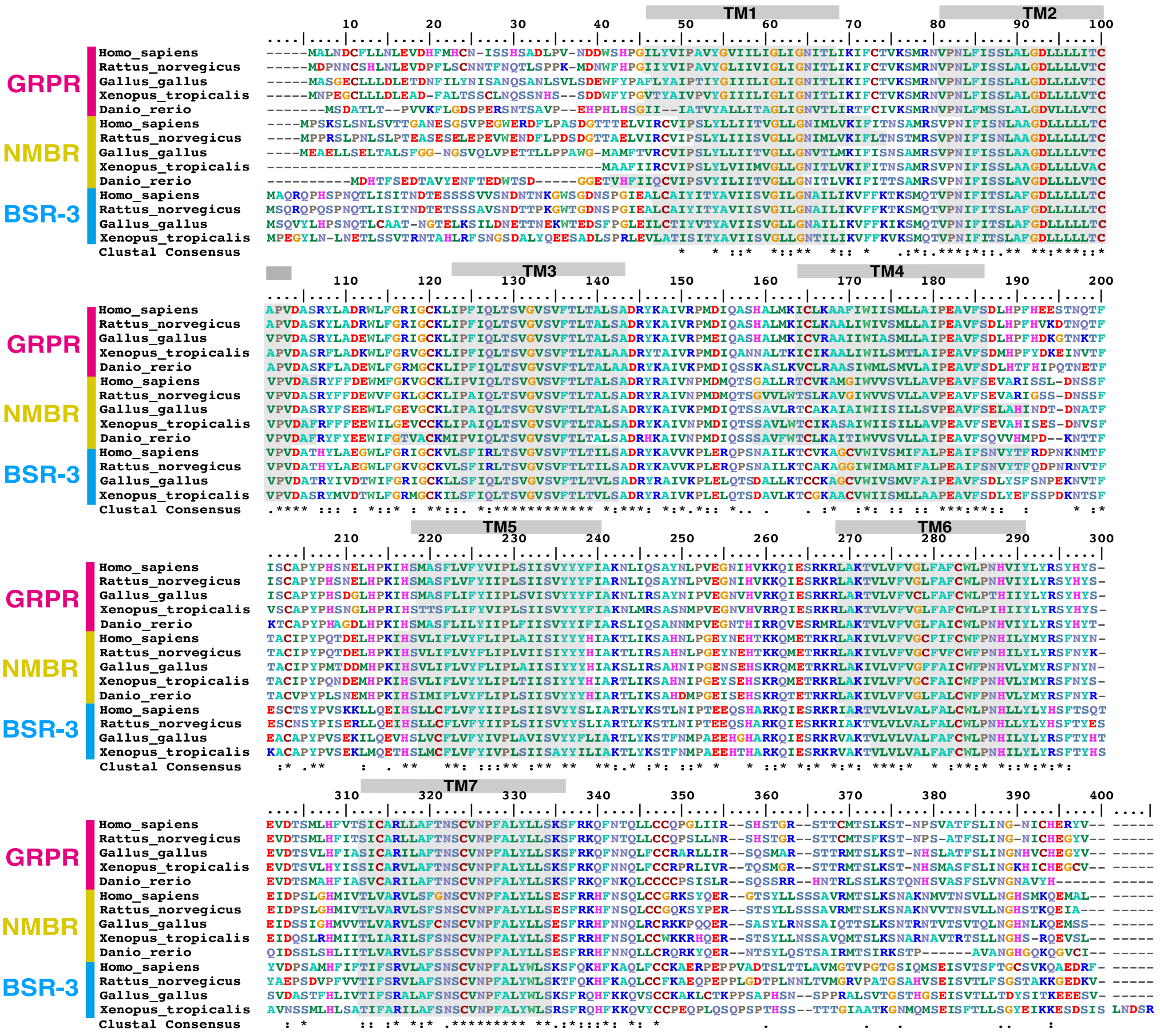

Supplementary Figure S2. Amino acid sequence alignment of gastrin-releasing peptide-preferring receptor (GRPR), neuromedin B-preferring receptor (NMB) and bombesin receptor subtype-3 (BRS-3). The seven transmembrane domains (TM1-7) in each sequence identified by TMHMM2.0 program are shaded in gray. IDs for the protein sequences used in this analysis are shown in Supplemental Table 1.

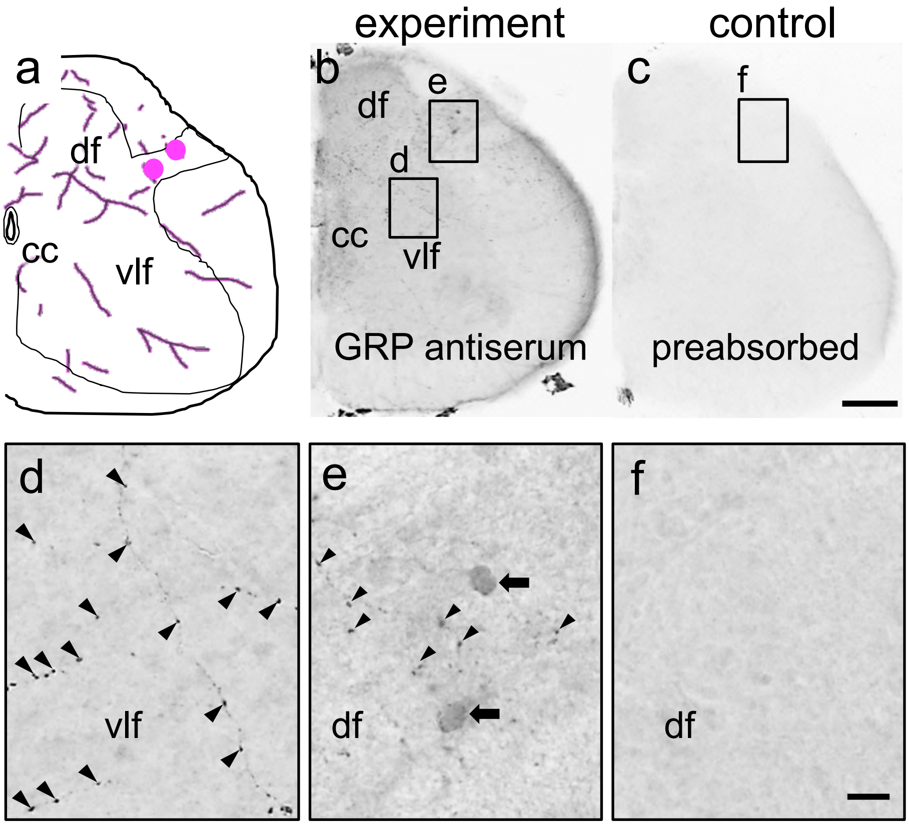

Supplementary Figure S3. Immunohistochemical identification of gastrin-releasing peptide (GRP) in the cervical spinal cords of *Xenopus*. (a) Schematic cross sections illustrating the distribution of GRP-like immunoreactivity. Immunoreactive cell bodies and fibers in *Xenopus* spinal cord are shown by magenta circles and magenta lines, respectively. GRP-immunoreactive cell bodies in the spinal cord were present in the dorsal field of spinal grey (df) (e; arrows). GRP-immunoreactive fibers and series of varicosities were found throughout the spinal grey (d and e; arrowheads). Preabsorbtion of the working dilution (1:2,000) of the primary GRP antiserum with a saturating concentration of GRP antigen peptide (neuromedin C; 50 mg/mL) overnight at 4° C before use eliminated the staining (c, f). Scale bars = 100 µm, 10 µm in enlarged images. For abbreviations, see Table 1.

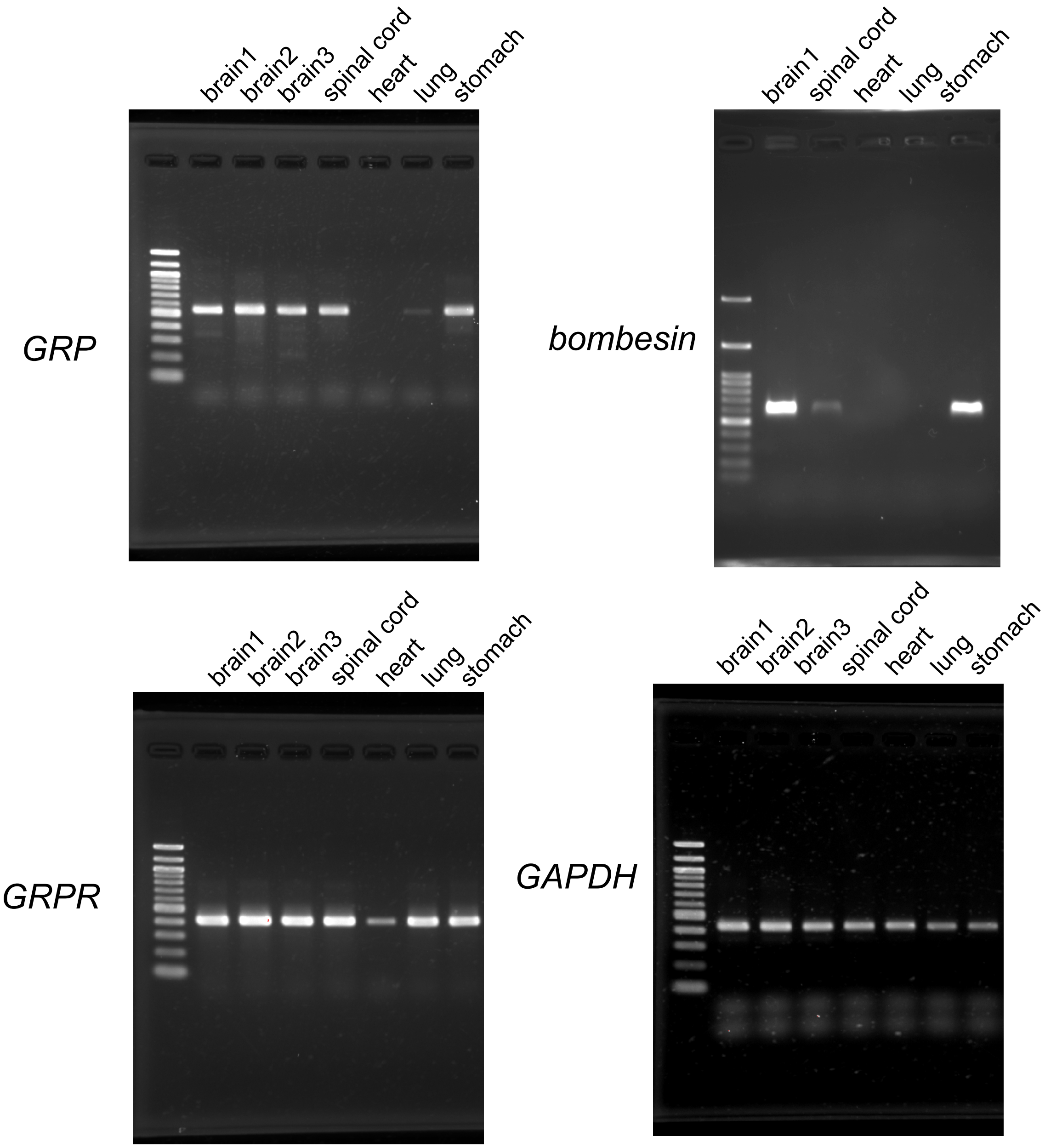

Supplementary Figure S4. Reverse transcription (RT)-PCR analysis of gastrin-releasing peptide (*GRP*), GRP receptor (*GRPR*) and *bombesin* mRNA expression in *Xenopus tropicalis*. RT-PCR for glyceraldehyde-3-phosphate dehydrogenase (*GAPDH*) was performed as the internal control.

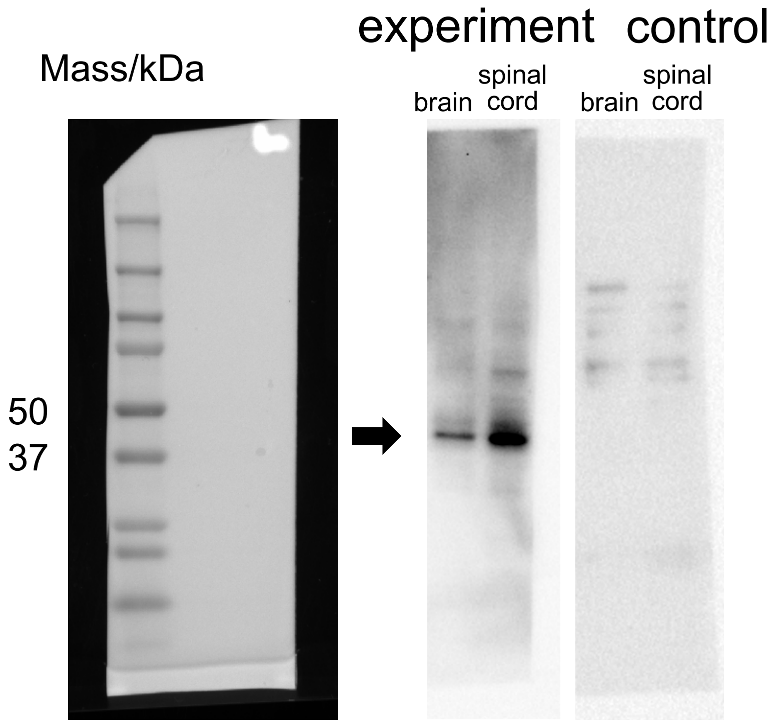

Supplementary Figure S5. Western immunoblotting of gastrin-releasing peptide receptor (GRPR). The number on the left indicates the molecular weight (kD). Extracts of protein from the *Xenopus* brain and spinal cord were transferred onto polyvinylidene difluoride membranes and probed with the rabbit polyclonal antibody against *Xenopus* GRPR (1:100,000). The antibody recognized a single band at the expected molecular weight of GRPR (~43 kDa) on a Western blot of the brain and spinal cord (experiment, arrow). Preabsorption of the antiserum with an excess of antigen peptides (50 µg/ml) eliminated the staining of the ~43-kDa protein band (control).

Supplemental Table 1. List of gene accession IDs.

species	GRP	NMB/Bombesin	GRPR	NMBR	BRS-3	endothelin receptor A	endothelin receptor B	drosha	GAPDH
Homo sapiens	NP_002082.2	NP_066563.2	NP_005305.1	NP_002502.2	NP_001718.1	NP_001948.1	NP_000106.1		
Rattus_norvegicus	NP_598254.2	NP_001102619.1	NP_036838.1	NP_036931.1	NP_690058.1				
Gallus_gallus	NP_001264829.1	NP_001072944.1	NP_989738.1	XP_426167.1	NP_989737.1				
Gekko_japonicus	XP_015261330.1	XP_015262375.1	XP_015273096.1	XP_015271810.1	XP_015279513.1				
Bombina orientalis	AAA51409.1	AAA48551.1, AAC59784.1, AAC59785.1	AAA79772.1	AAA79773.1	AAA91102.1				
Bombina variegata		CCG89208.1, CCE39585.1, CCE39584.1, CCG89209.1							
Nanorana_parkeri	XP_018425967.1	XP_018423141.1	XP_018413151.1	XP_018426182.1	XP_018410555.1				
Rana_catesbeiana		ACO51610.1							
Rana_pipiens		AAA49533.1							
Phyllomedusa_sauvagii		AAB32787.1, AAB32788.1							
Alytes_maurus		AFK29910.1							
Xenopus laevis	XP_018082396.1, XP_018098404.1	XP_018108353.1, NP_001079342.1	XP_018101519.1, XP_018104192.1	XP_018118724.1	XP_018087120.1, XP_018088621.1				
Xenopus tropicalis	XP_017946323.1	XP_004916033.1	XP_002938295.1	XP_012818691.2	XP_002932668.1			XM_031903197.1	XM_012966386
Microcaecilia_unicolor	XP_030047951.1	XP_030046218.1	XP_030055558.1	XP_030050971.1	XP_030065764.1				
Rhinatrema_bivittatum	XP_029435312.1	XP_029431696.1	XP_029457598.1	XP_029452772.1	XP_029463853.1				
Oryzias_latipes	XP_004074762.1	XP_004067328.2	XP_004081559.1, XP_023809111.1, XP_023806852.1	XP_020570338.1	not found				
Danio_rerio	NP_001154822.1	NP_001122228.1, NP_001076391.1	XP_695673.2	XP_690325.2	not found				
Lepisosteus_oculatus			XP_006639277.1	XP_006643031.1	XP_006633054.1				
Latimeria_chalumnae			XP_005998813.2	XP_006013596.1	XP_005990438.1				
Callorhinchus_milii	NP_001279882.1	XP_007906262.1	XP_007889883.1	XP_007904546.1	not found				
Rhincodon_typus	XP_020388898.1	XP_020371162.1	XP_020367369.1	XP_020375552.1	not found				
Branchiostoma_floridae			XP_002590601.1, XP_002590596.1						
Octopus_bimaculoides			XP_014782347.1						
Drosophila_melanogaster			NP_610199.2, NP_611241.2						

Abbreviations: GRP, gastrin-releasing peptide; GRPR, GRP-preferring receptor; NMB, neuromedin B; NMBR, NMB-preferring receptor; BRS-3, bombesin receptor subtype-3; GAPDH, Glyceraldehyde 3-phosphate dehydrogenase.

Supplemental Table S2. Primer sequences for cloning, reverse transcription-PCR, and real-time quantitative PCR.

Gene product	Forward primer (5'-3')	Reverse primer (5'-3')
Cloning		
GRP	TCTGACTGCAGACACATCATC	TTTGTGGAGCGGAAACCTCTG
GRPR	GAGGCTAACCCTGAATCTGATTACT	TAGGTGCTTTGTAGGTAGGTCAATG
Reverse transcription-PCR		
bombesin	ATGTCGGCTGTGCCTCTTAC	TCTTCGCTACTTCTGAGACG
GRP	TCTGACTGCAGACACATCATC	TTTGTGGAGCGGAAACCTCTG
GRPR	ATTGCGAAGAATTTGATGAGGAGTG	GTGGTTAGTGCTTTTGAGAGAAGTC
GAPDH	TTCACTGCCACCCAGAAGA	GTGGCTGTAGCCACACTCG
Real-time quantitative PCR		
GRP	CCCAGCAACACACAGAACCG	GCTCGCTCCACTCCCTCAT
GRPR	GTCCATGACTCTCGCCATCC	ATGGATTCTGGGGTGCAGTC
Drosha	TTACAGACCGCTGTTTGCTG	CAATTCGAGAGGGAGTTTCG

Abbreviations: GRP, gastrin-releasing peptide; GRPR, GRP receptor.

Supplemental Table 3. Primary antibodies used in this study.

Abbreviations: RRID, Research Resource Identifier; GRP, gastrin-releasing peptide; GRPR, GRP-preferring receptor.

Antigen	Description	Source, host species, cat#, or code#	Working dilution	RRID
GRP	Synthetic peptide mapping at 20–29 amino acids (GSHWAVGHLM) of rat GRP	AssayPro, rabbit polyclonal, 11081-05015	1:2,000	AB_2571636
GRPR	Synthetic peptide mapping at 195–208 amino acid residues (CAPYPHSNGLHPRI) of *Xenopus* GRPR	Generated by our laboratory, rabbit polyclonal, XGR14	1:100,000	AB_ 2832951
